# Advances in understanding infections caused by the basal fungus *Mucor*

**DOI:** 10.1371/journal.ppat.1011394

**Published:** 2023-06-01

**Authors:** José T. Cánovas-Márquez, Carlos Lax, Ghizlane Tahiri, Eusebio Navarro, Francisco E. Nicolás, Victoriano Garre

**Affiliations:** Departamento de Genética y Microbiología, Facultad de Biología, Universidad de Murcia, Murcia, Spain; Vallabhbhai Patel Chest Institute, INDIA

## Overview

Mucormycosis represents a group of infections with different organ manifestations caused by some species of the fungal order Mucorales. Historically, this disease has received limited attention due to its low worldwide prevalence, but the Coronavirus Disease 2019 (COVID-19) pandemic has led to an increase in the incidence of fungal infections, including mucormycosis, raising concern about their risks [1,2]. The prevalence of mucormycosis has increased by up to 50 times the previously recorded maximum [3], highlighting the need to better understand this disease [4]. Mucormycosis is an opportunistic infection that affects individuals with predisposing factors, including impaired immune function caused by different reasons, diabetes, and traumas in immunocompetent individuals [5]. The mortality rate is alarmingly high, with an overall rate exceeding 40% and approaching 100% in disseminated infections [6,7]. This high mortality rate is exacerbated by the intrinsic resistance of the causative agents to nearly all available antifungal drugs [8].

To understand the pathogenesis and antifungal resistance of Mucorales, researchers need to study models that are amenable to genetic manipulation, which faces the genetic intractability of these fungi. One outstanding exception to this general rule is *Mucor* species, particularly *Mucor lusitanicus*, which has the most comprehensive repertoire of molecular genetic tools for studying gene function [9]. Despite ranking third among the causative agents of mucormycosis, after *Rhizopus* and *Lichtheimia* species [6], the adoption of *Mucor* as a model to study mucormycosis has allowed researchers to study the function of putative virulence factors by the generation of knockout mutants. Some of the functional analyses have served to corroborate the results obtained in works employing causative models less willing to genetic modification, making the findings widespread throughout Mucorales, a critical aspect in the design of broad antifungals to combat mucormycosis. The contribution of these functional studies in *Mucor* to the advance in the understanding of processes associated with the infection is described in this Pearls mini-review.

## 1. *Mucor* spores survive the innate immune attack

**Fig 1:**
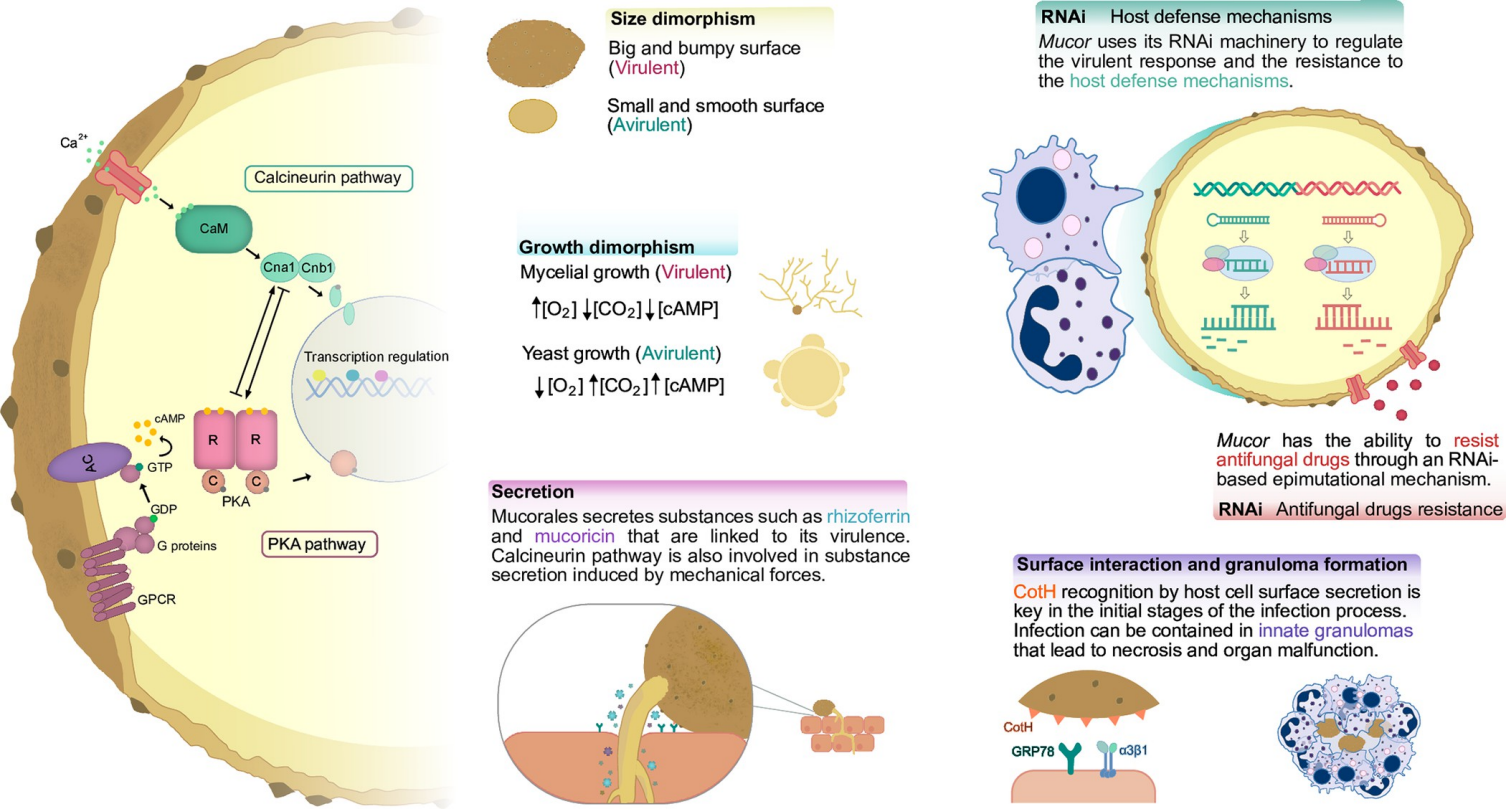
Molecular mechanisms involved in mucormycosis. Both calcineurin and PKA pathways are intricate and regulate key virulence determinants such as spore size dimorphism, growth dimorphism, phagosome maturation arrest, and substance secretion with direct targets and through transcriptional regulation. The posttranscriptional gene regulation orchestrated by RNAi is also decisive in the defense against host mechanisms and acquired antifungal drug resistance. During the infection process, the surface CotH proteins and their interaction with the host cell receptors facilitate the infection. Spores can be contained in structures constituted by innate immunity cells (innate granulomas), although the mechanisms leading to this process remain to be elucidated. AC, adenylyl cyclase; CaM, calmodulin; cAMP, cyclic adenosine monophosphate; GDP, guanosine diphosphate; GPCR, G-protein-coupled receptor; GTP, guanosine triphosphate; PKA, protein kinase A; RNAi, RNA interference.

Individuals with impaired innate immunity are at risk of developing mucormycosis. Studies of this first line of defense have helped identify key mechanisms involved in the pathogenesis of Mucorales and offer potential molecular targets for new therapeutic interventions. Analysis of *Rhizopus* invasiveness revealed that tissue invasion by Mucorales is mediated by CotH, which are cell surface proteins similar to bacterial proteins of the same name that are involved in spore coat formation [10]. The fungal proteins function as invasins that interact with specific host cell–type receptors (Fig 1), depending on the site of infection [11]. The feasibility of generating knockout mutants in *M*. *lusitanicus* has shown that at least 2 (*cotH3* and *cotH4*) of its 17 *cotH*-like genes are required for full virulence in a diabetic ketoacidosis (DKA) mouse model [12]. This work foregrounds the previous observations on the common high gene copy number in Mucorales [13,14], which could result in functional redundancy, an important aspect to be considered when designing therapies.

Once spores overcome physical barriers, macrophages and neutrophils are quickly recruited to the infection site. Despite these cells, mainly macrophages, phagocytizing the spores, they often fail to kill them, indicating the presence of spore-associated mechanisms to resist the attack. Several of these mechanisms have been described in Mucorales, suggesting that they have repeatedly evolved to survive natural environmental predators such as amoebae [15]. *Rhizopus* species use melanin on their spore surface to block phagosome maturation through inhibition of LC3-associated phagocytosis [16], while *Rhizopus microsporus* isolates interplay with the bacterial endosymbiont *Ralstonia pickettii* to secrete factors that protect fungal spores from phagocytosis and clearance by human macrophages [15]. *Mucor* species also induce phagosome maturation arrest, but by a different mechanism that depends on the calcineurin signal pathway [17]. In addition, *M*. *lusitanicus* spores display an intricate gene response induced by phagocytosis to allow germination within the phagosome and survive its hostile environment. Functional analysis of genes involved in this response identified 2 transcription factors, Atf1 and Atf2, as the major players in the response [18]. In this context, in vitro studies with macrophage cell lines and in vivo zebrafish infections suggest that *Mucor* spores can induce apoptosis in macrophages [17,19]. Although the mechanism that triggers this response in macrophages remains unknown, it is linked to the spore size dimorphism shown by *M*. *lusitanicus* in which the larger spores, but not the smaller ones, germinate inside macrophages [20].

In addition to spore engulfing, neutrophils and macrophages recruited at the site of infection form a cluster of phagocytes around the viable spores resembling an early innate granuloma (Fig 1). These structures have been observed in both *M*. *lusitanicus* and *Rhizopus arrhizus* and probably help to prevent fungal dissemination. However, these spores in the granuloma still maintain their viability and could lead to latent mucormycosis and disease reactivation under favorable conditions, such as immunosuppression [21,22]. Despite the advances in understanding the resistance of Mucorales to innate immunity, more effort is needed to have a clear picture of the mechanisms that could become drug targets to combat the infection.

## 2. *M*. *lusitanicus* RNAi mechanisms drive a genetic response to phagocytosis

The RNA interference (RNAi) mechanisms of *M*. *lusitanicus* perform a subtle control of endogenous gene expression [23]. Among the many processes regulated by the silencing pathways, recent investigations have focused on virulence. The pathogenic potential is a multifactorial trait, and the RNAi mechanisms orchestrate a genetic regulation layer that allows the fungus to adapt to environmental changes, as those found during host infection (Fig 1) [24]. Two antagonistic RNAi pathways are responsible for this refined regulation. On the one hand, the canonical RNAi mechanism uses small interfering RNA (siRNA), produced by Dicer enzymes, to guide the Argonaute-1 protein (Ago-1) to degrade the target transcripts. Analysis of the genes regulated by this pathway that showed differential accumulation of small RNAs (sRNAs) between virulent and avirulent pathotypes of *M*. *lusitanicus* revealed that the down-regulation of *wex1*, which codes for an exonuclease, is essential for virulence [25]. In the same research, 74 additional genes with a similar transcriptomic profile were identified, pointing to a key role of the canonical RNAi in virulence. On the other hand, the noncanonical RNAi pathway (NCRIP) degrades specific transcripts using the unusual catalytical activity against single-stranded RNA of the RNase-III R3B2 [26]. The transcriptomic analysis of NCRIP-defective mutants unveiled that the pathway represses both the genetic response launched during macrophage phagocytosis and the canonical RNAi [24]. Furthermore, the mutants in the NCRIP mechanisms exhibit both an increased resistance to oxidative stress and enhanced canonical RNAi functioning, but a reduced virulence in murine models [24]. These results highlight the importance of proper functioning of both RNAi mechanisms for an accurate response of *M*. *lusitanicus* during host infection, making the RNAi mechanisms a promising target for developing effective treatments against mucormycosis.

## 3. *Mucor* serves as a model to understand drug resistance in Mucorales

Clinicians have limited treatment options for Mucorales infections as these fungi show inherent resistance to most antifungals used in clinical settings, apart from amphotericin B. While the mechanisms behind this resistance are not yet fully understood, several studies on *Mucor* have shed light on them. A pivotal research found a conserved single amino acid substitution in the target for azoles (lanosterol 14a-demethylase CYP51 F5) that may be responsible for Mucorales’ innate resistance to short-tailed triazoles [27]. Gene duplication, a frequent situation in Mucorales [13,14], of the target gene and the calcineurin pathway contribute to the intrinsic resistance to the echinocandin micafungin [28]. Also, gene duplication of multidrug transporters may be behind the Mucorales antifungal resistance, as deletion of 2 out of 8 *M*. *lusitanicus* genes encoding putative ABC transporters of the pleiotropic drug resistance transporter subfamily only slightly increased susceptibility to some azoles [29].

In addition to genetic mechanisms to resist antifungal drugs, *Mucor* has evolved a nongenetic RNAi-based mechanism that generates transitory drug-resistant epimutants. This epimutational RNAi pathway was discovered in a search for the mechanisms underlying resistance to the antifungal drug Tacrolimus (FK506), which led to the isolation of drug-resistant epimutants that posttranscriptionally silenced the expression of the drug target gene (Fig 1) [30]. The mechanism works broadly in *Mucor*, as epimutants were also isolated showing a transient resistance to another antifungal agent, 5-fluoroorotic acid (5-FOA), by silencing the genes encoding the enzymes that convert 5-FOA into the active toxic form [31]. Interestingly, the rate of epimutation increases after host infection, especially in strains recovered from the brain, which is the most frequent target organ in Mucorales infection [32]. The epimutation mechanism could have a wider role beyond conferring drug resistance, enabling the fungus to adapt rapidly and temporarily to the challenges encountered during infection.

## 4. *Mucor* secretes substances to facilitate infection

Pioneer observations on the damage of human umbilical vein endothelial cells (HUVECs) by dead cells and hyphal debris from *Rhizopus* drove to the isolation of mucoricin, a hyphal-associated and secreted/shed toxin that is structurally and functionally similar to ricin [33]. This small protein induces necrosis, apoptosis, vascular permeability, and inflammation, and it is required for full virulence of *Rhizopus* in mice [33]. Interestingly, genes coding for this toxin are highly conserved across the genomes of several pathogenic mucoralean species, including *Mucor* (Fig 1), which turns it into a central player in the pathogenesis of mucormycosis. Other work has revealed that *Rhizopus delemar* secretes toxin-like peptides with immunomodulatory activities in conditions mimicking human infection [34]. Additionally, some species may have bacterial endosymbionts that contribute to the secretion of factors that facilitate infection, as seen in the interaction between *R*. *microsporus* and *R*. *pickettii*, described above [15].

Mucorales also secrete molecules to uptake essential nutrients for infection. There are 2 main mechanisms of iron uptake that are involved in Mucorales pathogenesis: the reductive high-affinity system and siderophores. The components of the first system, located in the cytoplasmic membrane, play a significant role in virulence in *Mucor* and *Rhizopus* [35]. Mucorales also secrete rhizoferrin (Fig 1), a polycarboxylated siderophore that is synthesized by a nonribosomal peptide synthetase encoded by *rfs* gene. The deletion of this gene in *M*. *lusitanicus* leads to lower mortality in mice, while its overexpression increases virulence, suggesting a role of rhizoferrin in mucormycosis [36]. Interestingly, the expression of *rfs* is induced by the oxidative metabolism, which is also associated with the filamentous growth and virulence of *Mucor* [36].

The secretion of molecules important for virulence can be regulated by environmental conditions, as shown by the tornadic shear challenge that induces a transient hypervirulent phenotype in *Mucor* and other clinically relevant Mucorales. Although the soluble factors released by stressed spores remain unknown, the calcineurin/Hsp90 axis is critical for increasing virulence in the infected host, but surprisingly, it is not directly involved in the secretion of these factors [37]. In addition, a comparative analysis of the genomes of virulent and avirulent pathotypes of *M*. *lusitanicus* identified 28 genes that code for putative extracellular proteins, several of which have unknown functions and appear only in the virulent strain. These genes are potential candidates for involvement in pathogenesis. While systematic mutation of these genes could be carried out in *Mucor*, only 2 have been deleted so far, revealing that a gene of unknown function is required for full virulence in mice [38].

## 5. Hyphal growth and oxidative metabolism is required for virulence

A fascinating characteristic of *Mucor* is its dual morphotype, either as a unicellular yeast-like organism or as a filamentous mycelium (Fig 1). Unlike most fungi that present only 1 morphotype, dimorphic fungi have evolved to maintain all the genomic information necessary to alternate between the 2 morphologies.

The dimorphism of *Mucor* determines not only its morphology but also the type of metabolism and virulence potential, with the hyphal growth being required for virulence [39]. Fermentable carbon sources, anaerobic conditions, and high cAMP tend to promote yeast growth, while oxygen fosters mycelial growth independently of the carbon and nitrogen sources (Fig 1). Regarding the molecular regulation of dimorphism, the calcineurin pathway is particularly significant in the yeast-hyphal transition (Fig 1). Thus, a mutation in *cnbR*, encoding the regulatory subunit of calcineurin, results in high protein kinase A (PKA) activity and yeast-locked morphology, even in the presence of oxygen, suggesting a connection between PKA and calcineurin in the control of dimorphism. Interestingly, lack of *cnbR* reduced virulence in *Galleria mellonella* [39], supporting that hypha is the virulent form.

Heterotrimeric G proteins positively regulate mycelial growth under low oxygen conditions through also the PKA pathway. The deletion of *pkaR1* or *gpb1*, which encodes one of the 4 regulatory subunits of PKA or the heterotrimeric G-beta subunit 1, respectively, results in increased yeast growth under low oxygen levels and reduced virulence in DKA mice [40]. On the other hand, a mutation in *adh1*, which encodes alcohol dehydrogenase 1, produces a monomorphic hyphal strain that lacks the ability to perform fermentative metabolism and shows higher virulence than the wild-type strain in mice [41]. All these data indicate that control of dimorphism promoted by oxidative metabolism is crucial for efficient pathogenesis in *M*. *lusitanicus*, with the hyphal form linked with a more virulent phenotype, partly due to enhanced tissue invasion.

A second dimorphism has been described in *M*. *lusitanicus*, which affects the size of the spores (Fig 1). This dimorphism is also linked to virulence with a positive correlation between spore size and virulence potential [20]. Calcineurin pathway regulates spore size because mutants lacking the catalytic subunit CnaA produce larger spores, and these are more virulent than the wild-type strain [39,42]. Similarly, deletion of *gpa11* and *gpa12* encoding heterotrimeric G-alpha subunits results in larger spores than the wild-type strain, but with similar virulence potential [42]. Both calcineurin and G proteins are interlocked at the transcriptional level, but more research is needed to unveil how they regulate spore size and virulence.

## Outlook

Effective control of mucormycosis requires a deep understanding of the molecular mechanisms involved in the critical steps of the infection, such as adhesion, invasion, and colonization, as well as those involved in the innate resistance of Mucorales. Over the past decade, there has been significant progress in characterizing the genes and mechanisms involved in Mucorales pathogenesis, with *Rhizopus* and *Mucor* serving as model organisms. Despite *Mucor*’s lower virulence compared to *Rhizopus*, it is important because it allows for the direct study of gene function. A decade of generating mutants in candidate genes has identified several that are required for full virulence, but none of them are essential, indicating that the pathogenesis of *M*. *lusitanicus*, and probably Mucorales in general, is a multifactorial character. Moreover, *Mucor*, in opposition to *Rhizopus*, shows dimorphism that is linked to virulence, highlighting the differences in pathogenesis among different species and genera of mucormycosis causative agents, an aspect that require our attention. The ability to study gene function in *Mucor* has also contributed to understanding of the mechanisms underlying both innate and acquired resistance to antifungal drugs. The incorporation of *R*. *microsporus*, which is highly virulent and amenable to genetic manipulation [43], as a model of mucormycosis is expected to further our knowledge about the disease and promote the development of new antifungals that overcome the natural antifungal resistance of Mucorales.
